# Ernst B. Haas, liberal nationalism and the double-edged nature of European identity

**DOI:** 10.1080/13501763.2024.2379443

**Published:** 2024-07-22

**Authors:** Theresa Kuhn

**Affiliations:** Department of European Studies, University of Amsterdam, Amsterdam, The Netherlands

**Keywords:** Nationalism, Collective identity, European integration

## Abstract

While Ernst B. Haas is recognized among European integration scholars as a founding figure of neo-functionalism, his later writing on nationalism is less known to European integration scholars. I contribute to this special issue by discussing Haas’ later work on liberal nationalism and by reflecting on its insights for the study of collective identities in the European Union today. In a nutshell, Haas expected that (1) national and supranational identities are inherently rational and the expression of a deliberate choice, (2) citizens shift their identities from the national to the European level due to utilitarian considerations, (3) nationalism is not per se destructive, but it comes in many moulds and can be the basis for the international community. I argue that Haas’ emphasis on rational and deliberate identity choice clashes with our current understanding of identity as implicit and subliminal. Moreover, his optimism is challenged by the rise of radical-right-wing parties who have successfully mobilized exclusive nationalist identities against European integration. On the other hand, Haas’ emphasis on the double-edged nature of collective identity is an important reminder that also European identity, while striving to overcome exclusive nationalism, can reify its exclusiveness at the supranational level.

## Introduction

Ernst B. Haas is predominantly celebrated as the founding figure of neo-functionalism. In his path-breaking book, *The Uniting of Europe,* Haas argued that European integration was a complex, self-reinforcing process that is predominantly driven by a coalition of supranational institutions and national interest groups that lobby for further integration. Integration in one policy area would lead to spillovers, facilitating integration in other policy areas (Haas, 1958, 1970). This book provided the basis of neo-functionalism, which was next to intergovernmentalism (Hoffmann, 1966) one of the two ‘grand theories’ of European integration that have dominated scholarly research and teaching about European integration for decades. As Rosamond (2005, p. 238) put it, *The Uniting of Europe* represents the founding moment of the field of what we now routinely term ‘EU studies’. Haas’ writing on the drivers of European integration has also found its imprint in European policymaking (Ruggie et al., 2005). Even today, more than seven decades after Haas formulated his expectations; his work is still being used to make sense of current developments in European politics (e.g., Brooks et al., 2023; Hooghe & Marks, 2019; Nicoli, 2020; Schimmelfennig, 2016).

Despite the initial huge influence of neo-functionalism on EU studies and international relations more generally, the bumpy development of European integration in the *Eurosclerosis* period, i.e., a period of stagnation in European integration in the 1970s and 1980s, put a major question mark on its optimistic expectation about self-reinforcing integration cycles (Haas, 1976). In this period, the state-centred assertions of intergovernmentalism (Hoffmann, 1966) received more empirical support. In fact, Haas (1976) himself acknowledged that the core assumptions of neo-functionalism were not met any more.^1^ It might be for these reasons that Haas returned to his original research interest, nationalism, in his later career.

Scholars studying the politics of European identity might not readily turn to Haas. After all, Haas’ early neofunctionalist scholarship (1958, 1970) of European integration is centered on elites and focuses on the triumph of reason over sentiment. To be sure, Haas defined political integration as a process that involves actors ‘shifting loyalties’ to the new political centre (Haas, 1958, p. 5). However, this shift in loyalties predominantly reflected rational cost–benefit calculations in response to functional pressures to integrate and ‘for the satisfaction of important expectations’ (Haas, 1958, p. 5). Haas deemed deeper changes of identity as highly unlikely, and collective identities played a minor role in neo-functionalism (Checkel & Katzenstein, 2009, p. 5). Moreover, in *The Uniting of Europe,* Haas focused on political leaders and did not consider changes in loyalties among the wider public. He did so because he deemed citizens to be ignorant of European integration, and because in the early days of European integration, the public virtually had no say in this process as European institutions were shielded from public scrutiny and representation (Haas, 1958, pp. 17–18; see also Kuhn, 2019).^2^ For all these reasons, neo-functionalism has little to offer for scholars working on mass European identity. Among the early theories of European integration, Karl Deutsch’s transactionalist theory (1957) lends itself much better to research on European identity as it addresses public opinion and identity change more heads-on by arguing that transnational interactions among ordinary people lead to a common ‘we feeling’ (Deutsch, 1954 [1970], p. 36).

In turn, questions of collective identity are much more prominent in Haas’ later writings on nationalism (Haas, 1997, 2000). This work was widely cited among scholars of nationalism (eg. Brubaker & Laitin, 1998; Hechter, 2000) and in research on rationalism in international politics (Milner, 1998). In contrast, it has received much less attention among European integration scholars.

In this article, I examine Haas’ later work on nationalism to ascertain what we can learn from it for the study of collective identities in an integrating Europe today (see also the contribution by Börzel, 2024). On the one hand, I argue that Haas’ emphasis on rational and deliberate identity choice does not bode well with our current understanding of identity formation which emphasizes its implicit and subconscious character (Cram, 2012). This helps explain why Haas’ optimistic expectations about liberal nationalism as a basis for supranationalism have not materialized. On the other hand, Haas’ discussion of the double-edged nature of collective identity is an important reminder that also European identity, while striving to overcome exclusive nationalism, can reify its exclusiveness at the supranational level. As the EU strengthens its external boundaries, and also far-right-wing politicians such as Giorgia Meloni increasingly embrace European cooperation and try to shape the EU according to their own nationalist ideas, this message is more lucid and important than ever before.

In what follows, I summarize Haas’ main argument on nationalism. I then discuss what Haas’ research tells us about the prospects of the international community and European identity and discuss to what extent current empirical research on European identity supports or undermines his expectations. I focus on the two volumes of *Nationalism, liberalism, and progress* (Haas, 1997, 2000), but also consider other contributions, such as *Beyond the Nation State* (Haas, 1964) and his articles on this topic in *International Organization* (Haas, 1970, 1976, 1986).

## Ernst B. Haas in defense of liberal nationalism

After decades of developing the central building blocks for neo-functionalist theorizing and providing food for thought for generations of EU scholars, students, and policy-makers, Haas returned to his earlier research on nationalism (Caramani, 2024). In fact, in *Nationalism, liberalism, and progress* (Haas, 1997, 2000), his research on nationalism took off where he left in the last chapter of *Beyond the Nation state* (Haas, 1964) forty years earlier.

According to Ruggie et al. (2005), Haas returned to the study of nationalism because he was disillusioned with the prospect of the international community and European integration. To be sure, Haas continued to searching for ‘international happiness’ (Haas, 1997, p. 18), as he put it, but he argued that international relations can only be understood once one knows how nation-states evolved and are held together (Haas, 1997, p. 22). He also posited that liberal nationalism would have the greatest potential to provide a basis for the international community (Haas, 1997, p. 19). In a nutshell, Haas posited that nationalism comes in different moulds, and that too much attention has been given to its negative and destructive aspects while the positive aspects of liberal forms of nationalism, such as rationality, modernity, and progress, have been largely ignored.

In *Nationalism, liberalism, and progress* (Haas, 1997, 2000), Haas presents and discusses with great attention to detail the historical trajectories of state- and nation-building of five ‘successful’ liberal nations (The US, UK, Germany, France, and Japan) in the first volume and focuses on a number of ‘newer’ nations (Iran, Egypt, India, Brazil, Mexico, China, Russia, and Ukraine) in the second volume. What is of most interest for the purpose of this article is the theoretical framework of both volumes, and in particular, the sections about nationalism and national identity.

### The mass politics of nationalism

In contrast to his earlier, elite focused work on neo-functionalism, Haas takes mass politics into account in his study of nationalism and recurrently makes explicit reference to questions of people’s collective identity. He defines a nation as a “socially mobilised body of individuals who believe themselves united by some set of characteristics that differentiate them [… ] from outsiders and who strive or create their own state” (Haas, 2000, p. 23). Echoing Benedict Anderson’s (1983) notion of ‘imagined communities’, Haas sees nationalism as a “feeling of collective identity that is experienced as mutual understanding among people who will never meet but who are sure they belong to a community of others just like them, and different from ‘outsiders’” (1997, p. vii).

Haas argues that whether nationalism has been successful depends on the level of *saturation* in society (Haas 1997): National sentiment (low saturation) is limited to a group of intellectuals who feel solidarity towards each other, while national ideology (medium saturation) is a political programme that is supported by a larger share of the population, and national myth (high saturation) is a commonly held shared understanding of what constitutes the nation (Haas, 1997, p. 43). This shared understanding can be based on religion, language, race or social status. If there is no consensus on what constitutes the nation, nation-building was not successful (Haas, 1997, p. 43). In such a situation, conflict looms large.

Haas posits that an important role in the development of liberal nationalism is played by social learning, which involves behaviour change and redefinition of means and aims based on new knowledge (Haas, 1997, p. 170). This echoes earlier work by Karl Deutsch who argued that transnational interactions and experiences would set off cognitive learning processes which in turn would lead to a common identity (Deutsch et al., 1957). However, the concept of social learning remains underdeveloped in Haas’ writing (Risse, 1998). While Haas goes to lengths to discuss how learning is different from mere adaption (Haas, 1997, p. 170), it remains unclear under which circumstances societies really learn rather than simply adapt, and what the underlying cognitive processes are.

### Nationalism as ‘an instrumental social construction’^3^

For Haas, nationalism is a byproduct of modernization, a way to make sense of and provide coherence in ever more complex societies that are undergoing rapid social change (Haas, 1970, 1997, p. 24). National identity, according to Haas, is ‘an instrumental social construction’, (Haas, 1997, p. 22): It is socially constructed through interaction and communication rather than being exogenously given, and it is an identity by choice and the result of a rational, instrumental cost–benefit calculation. Nationalism, in Haas’ understanding, is a tool to avoid conflict in a time of social unrest by unifying rivalling interpretations of who is ‘we’, and what it is based on. According to Haas, “nationalism can hold a society together while people are being buffeted by the strains of modernization” (Haas, 1970, p. 710).

Echoing his writing on neo-functionalism, Haas emphasizes *Nationalism, liberalism, and progress* the triumph of reason over sentiment. According to Haas, rational actors decide to move allegiances based on instrumental cost–benefit calculations. He writes: “I assume the dominance of instrumental motives among actors: they choose to act as nationalists for instrumental reasons” (Haas, 1997, p. 36). However, in contrast to his neofunctionalist writings (and potentially informed by intergovernmentalism), he now conceives of interests as being aggregated at the national level, and nation-states, rather than transnational interest groups or supranational organizations, as main actors in international politics.

Haas goes even further by emphasizing that also ethnic nationalism can be the result of rational cost–benefit calculations rather than the mere expression of affective allegiance to an ethnic group. For example, he argues that “ethno-nationalists, being modern and sophisticated people, are easily bought off” (Haas, 1997, p. 42). From this perspective, ethnic conflicts could be relatively easily solved by providing economic incentives to overcome ethnic nationalism.

### Liberal nationalism and the prospect of European identity

What does Haas think about the prospect of supranational identity in Europe? In his 1995 article on nationalism, Haas writes that a world made up of successful nation-states is “probably an inherently bellicose world” (Haas 1995, 507), especially if these states are not based on liberal democracy. The only solution is what he calls international rationalization, i.e., the formation of a global or regional state in the making, and he only deems that possible, if at all, in Western Europe. In *Nationalism, liberalism, and progress,* Haas takes a more optimistic stance as he sees international rationalization as a likely outcome. He expects many nation-states to ‘outgrow’ their statehood (Haas, 1997, p. 59): In an increasingly globalized market, citizens in advanced industrialized societies expect ever higher standards of living which states cannot provide unilaterally. Hence, *based on instrumental calculations*, citizens become less attached to the nation-state and move their allegiances to the supranational level. In turn, nation-states engage in multilateral bargains and institutions to keep their constituencies happy (1997, p. 60). In other words, they try to stay in power by giving some power away. There are clear parallels between this expectation with earlier neofunctionalist arguments. Importantly, according to Haas, national and European identity does not necessarily exclude each other. As long as people hold liberal views of nationalism, they can accommodate a national and a European identity.

Hence, from a Haasian perspective, political identities are inherently instrumental (Haas, 1997, p. 22). European identity develops if European integration provides more benefits to citizens than the nation-state. This perspective thus predicts that collective identity goes hand in hand with the policy output and performance of European institutions. During the euro crisis, for example, European identity should be weaker cognition in economically stable times.

### The two sides of nationalism

Haas (1997, p. vii) takes issue with the dominant ‘dark view of nationalism’ which portrays nationalism as per definition illiberal and destructive, leading to ethnic conflict, aggression, and even genocide. While Haas contends that nationalism can have many negative consequences, he advocates for an understanding of liberal nationalism that emphasizes its modern, rational and unifying aspects. In Haas’ view, national and European identities do not necessarily exclude each other, if people hold liberal views of nationalism, they can accommodate a European in addition to a national identity. According to Haas, liberal nationalism is the most promising vehicle towards human progress (Haas, 1997, p. 24), even more promising than regional integration or global governance (Ruggie et al., 2005, p. 289). In a period that was marked by devastating nationalist conflicts across the European continent – the wars in ex-Yugoslavia, the Nagorno-Karabakh Conflict between Azerbaijan and Armenia, the ‘Troubles’ in Northern Ireland and terrorism by the Basque separatists, to name but a few – this might have seemed quite provocative as nationalism was generally understood as the opposite to liberalism (Börzel, 2024, p. 8).

In fact, Haas highlights that nationalism can come in many shapes and sizes, also among liberal democracies, and that it is not per definition destructive nor hostile towards outgroups. Many states actively engage in nationalist ideology, often with more integrative elements. Haas does not follow the prominent distinction into (‘good’) civic vs (‘bad’) ethnic nationalism, i.e., whether nationalism is based on civic or ethnic criteria of inclusion (Ruggie et al., 2005, p. 289). Rather, he distinguishes between liberal and illiberal forms of nationalism, and within liberal nationalism he distinguishes between revolutionary and reformist (Haas, 1997, p. 22ff). Haas puts all his bets on liberal nationalism, and he defines liberalism as a form of governance based on democratic decision-making processes that represent major societal interests through elections and public deliberation (Haas, 1997, p. 20, for a more elaborate discussion, see also Börzel, 2024).

It is interesting to note the differences and parallels between Haas’ earlier neofunctionalist work on regional integration and his later work on liberal nationalism. In the latter, Haas echoes his neofunctionalist approach by conceptualizing human actors as rational and deliberate, and he continues to be optimistic about the potential for human and societal progress. It seems what has changed most throughout Haas’ career is the emphasis on the political level of aggregation. While in his neofunctionalist work on regional integration, Haas put all his bets on supranational institutions, his later work focuses on the national community as the main agent of change. Moreover, while Haas found it safe to ignore citizen identities and preferences in his early work on regional integration, mass politics played a more central role in his work on liberal nationalism.

## Do Haas’ expectations of liberal nationalism stand the test of time?

Having reviewed Haas’ writing on liberal nationalism, I now turn to the question of how his expectations have panned out in reality, and to what extent a re-engagement with his scholarship could be useful for our understanding and analysis of collective identities in an integrating Europe today.

### Defining collective identity

Collective identities structure how we see ourselves in relation to social groups (Kohli, 2000). Scholars often draw on insights from social identity theory (Tajfel, 1982) and define social identities as people’s understanding as members of a group and the emotional weight linked to this membership. These identities are socially constructed, in other words, identities are not given at birth but are being built through interaction and socialization with other members of the collective and with ‘Common Others’ which help to define the group boundaries (Risse, 2010). In line with social identity theory, collective identities have multiple dimensions. The cognitive dimension relates to whether people *identify as* – or see themselves as – members of a collective. The emotional dimension captures the extent to which individuals also *identify with* the collective and their emotional attachment to it (Cram, 2012).

Next, the evaluative dimension relates to judgments about the collective in relation to a ‘Common Other’, i.e whether is it seen as superior to other groups and leads to outgroup bias and discrimination. Related to this are criteria that define the collective, i.e., the content on which identity is built (Abdelal et al., 2006). Borrowing from a well-established distinction in nationalism research (Kohn, 1944), scholars differentiate between ethnic/cultural and civic forms of European identity (Bruter, 2003; Schlenker, 2013). Ethnic/ cultural understandings of European identity are expected to be more exclusive and hostile towards non-European outgroups (König, 2023; Risse, 2010; Schlenker, 2013).

European identity does not necessarily replace national identity (Huddy & Del Ponte, 2019), as many people conceive of European identity as supplemental to their national one (Hooghe & Marks, 2009, Risse, 2010). And in fact, it is one of the big achievements of the EU that it was able to build a narrative of a supranational identity that can go hand in hand with national identity (Kuhn, 2023).

In the remainder of this section, I discuss to what extent Ernst B. Haas’ main expectations on liberal nationalism and collective identities in an integrating Europe resonate with current research on European identity and have been confirmed by empirical studies. In particular, I focus on three main assertions that I have discussed above: Haas expected that (1) national and supranational identities are inherently rational and the expression of a deliberate choice, (2) citizens shift their identities from the national to the European level due to utilitarian considerations, (3) nationalism is not per se destructive but can be the basis for the international community.

### Rationality as a source of collective identity

Turning to Haas’ emphasis on rationality as a source of collective identity, it evidently clashes with the state of the art in theoretical and empirical research on European identity. To be sure, the argument that citizens move their allegiances to the supranational level if supranational institutions can provide more benefits than the state has been brought forward by Deutsch et al. (1957, p. 85) and has clear parallels with Catherine de Vries’ benchmarking argument that sees EU support as the result of a rational comparison between the domestic status quo and the EU (De Vries, 2018). However, it is important to highlight that benchmarking theory does not refer to collective identity, but rather to utilitarian support for European integration and EU membership which is per definition more rational. Most importantly, Haas’ understanding of a deliberate identity choice based on instrumental calculations does not rhyme well with other theoretical perspectives that see identities as less deliberate and more implicit and subconscious^4^ (Cram, 2012). Easton (1975), for example, sees diffuse support for political systems (a concept which has many parallels to collective identity) as stable and rather insensitive to short-term changes in policy outcomes. As Easton posits (1975), diffuse support serves as a reservoir of support in times when there is little (rational) reason for specific support.

While Haas expected collective identities to reflect rational choice and functionality, postfunctionalist theory (Hooghe & Marks, 2009) shows that reality is more complex as collective identities do not change as easily. Postfunctionalism asserts that precisely the mismatch between rational pressures for regional integration and sticky national identities is at the heart of the conflict over European integration and determines the speed and shape of further institution building (Hooghe & Marks, 2009, 2018). This is also what empirical survey research suggests: A large body of research has shown that collective identity and preferences for European integration do not necessarily align with economic costs and benefits (Reinl et al., 2023; Risse, 2010). Most prominently, the Brexit referendum outcome and Britain’s subsequent exit from the European Union cannot be explained by rational choice theory as it was clearly not aligned with economic interest (Hooghe & Marks, 2018). The *Remain* campaign had focused on instrumental reasons for staying within the European Union (‘Britain stronger in the EU’) but ultimately could not win over the hearts and minds of British voters. They were more swayed by the *Leave* campaign’s focus on an ethnic threat by immigration (Hobolt, 2016, p. 1263), cultural values (Dennison et al., 2020), and concerns about the ethnic composition of the British population (Kaufmann, 2019).

**Figure 1.**
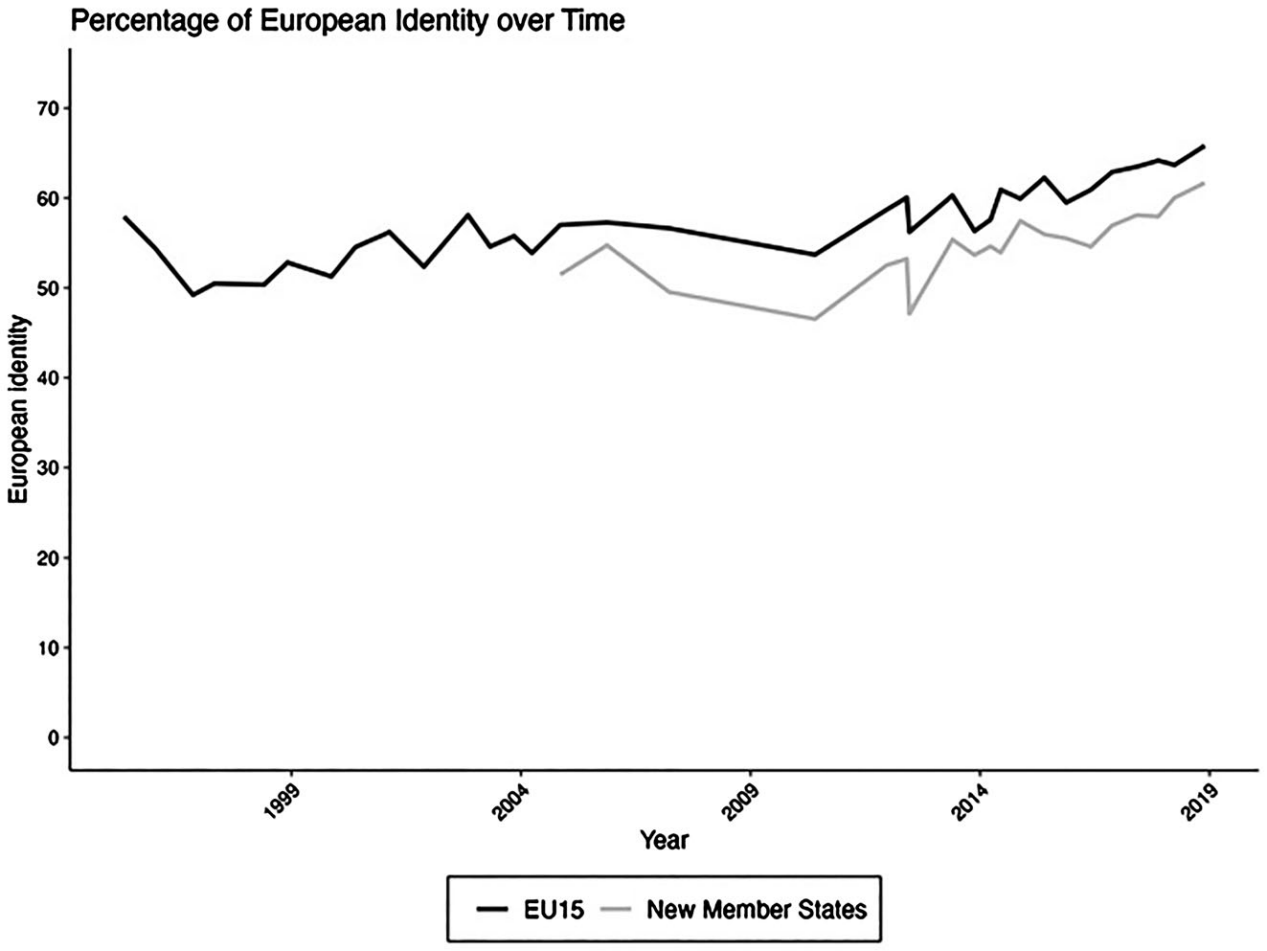
European identification over time. Source: own calculation based on Eurobarometer surveys 1995–2019.

On the other hand, from a rational perspective, European identity should have decreased in the period of the first European polycrisis (Zeitlin et al., 2019): The eurozone crisis and subsequent economic bailouts, the failure to handle the migration influx and distribution migrants across member states, and Britain’s decision to leave the UK all put a big question mark on the EU’s capability to deal with these challenges, and may have suggested that European integration was the problem rather than the solution. However, empirical evidence shows that European identification increased during the first and the second polycrisis (see Figure 1; Nicoli et al., 2024). This suggests that European identity is also an expression of feeling to be part of a community of fate. Also beyond these crisis periods, people seem to care more about ‘who we are’ than ‘what we get’, and this has important implications for political behaviour. A large literature on voting behaviour has shown that cultural issues have become increasingly important in structuring political conflict, and the issue of European integration is central to it (Hooghe & Marks, 2009, 2018; Kriesi et al., 2008).

Equally, Haas’ conception of identity as a deliberate choice clashes with current research on collective identities which underlines its implicit and subliminal nature (Abdelal et al., 2006; Cram, 2012). Research strongly suggests that European identity is being built in ‘banal’ ways by establishing the EU as a social and political fact that makes people become aware of European integration in their daily lives (McNamara, 2015). European institutions have engaged in so-called identity programmes (Kaina & Karolewski, 2013; Saurugger & Thatcher, 2019) to give the EU a ‘meaningful presence’ in people’s lives (Cram, 2012, p. 72; McNamara, 2015), which in turn leads them to adopt a European self-identification (Risse, 2010).

European identity formation predominantly happens through two main mechanisms which broadly relate to two central approaches in social theory, the structural and the culturalist approaches (Recchi 2015). First, according to the structural approach, European integration provides opportunities for increased cross-border interactions among citizens. These interactions are expected to trigger learning processes which in turn foster a common identity (Deutsch et al., 1957; Kuhn, 2015). Research shows that highly transnational individuals are indeed more likely to feel European (Fligstein, 2008; Kuhn, 2015). The second mechanism linking European integration to identities relates to exposure to common norms and symbols, which is expected to set forth socialization processes, i.e., ‘inducting actors into the norms and rules of a given community’ (Checkel, 2005: 804). Socialization happens either directly in institutions or organizations (Checkel, 2005, Hooghe, 2005), or more broadly through exposure to common symbols and myths in the public sphere (Cram & Patrikios, 2015; McNamara, 2015; Negri et al., 2021). Hence, in contrast to what Haas asserted, citizens do not deliberately choose their political identities. Rather, these identities are the result of a complex process of social learning through interaction and socialization through exposure to common norms and symbols.

### Shift towards supranational identities

Next, empirical evidence partly confirms Haas’ expectation that people would move their identities from the national to the European level, but the question remains whether the glass is half full or half empty. Survey research suggests that a feeling of European identity in addition to national identity has been slowly growing over the past few decades (Bergbauer, 2018; Hadler et al., 2021; Lutz et al., 2006; Negri et al., 2021; Schröder et al., 2024). Since the early 1990ies, Eurobarometer has been repeatedly surveying respondents across Europe on the cognitive dimension of European identity by asking them (with slight variations in the wording)^5^ whether they see themselves as (also) European or only national. Figure 1 shows that for the period that we have records, at least half of Europeans see themselves as (also) European. The pattern for countries having joined the EU in 2004 and onwards is very similar to the EU-15 albeit at a slightly lower level. Importantly, and perhaps surprisingly, this share has been slowly growing over the past decade. It is plausible that external threats such as the COVID-19 pandemic and Russia’s war on Ukraine have nurtured a feeling of common fate. What is more, most Europeans hold a ‘European identity lite’ (Risse 2015): this means, they still see themselves first and foremost members of their national community, and their European allegiance is only secondary. This slowly moving trend towards supranational identity has been confirmed in studies using the World Values Survey (Jung, 2008; Norris, 2000) and ISSP (Hadler et al., 2021).

Lutz et al. (2006) use Eurobarometer data and advanced statistical methods to project the development of European identity into the future. They expect that by 2030, one out of four Europeans in the age group 30–44 will identify as European. This expected increase in European identity is likely due to socialization and exposure to European values and norms. Younger generations have been socialized in a more Europeanized society, they take the EU, its institutions, and its impact on their daily live and live chances for granted (Rekker, 2018; Shorrocks & de Geus, 2019; but see Jung, 2008 and Schröder et al., 2024 for a competing view).

Hence, Haas’ expectations of shifting identities towards the supranational level find some support in current research and empirical evidence, especially when considering European identity ‘lite’: (Risse 2015). However, it is important to point out that a significant share of Europeans continues to feel exclusively national, and European identity is unevenly distributed across society. In addition to the generational differences discussed above, there are significant socio-economic differences in European identity. People with lower levels of education and those in lower-status jobs tend to be less likely to feel European and more eurosceptic (Fernández & Eigmüller, 2018, Kuhn et al., 2021). While these differences reflect self-interest (Fernández et al., 2023), they develop early in the life course and are primarily a result of different exposures and experiences with Europe (Deutschmann et al., 2018; Kuhn, 2015; Shorrocks & de Geus, 2019), parental socialization (Kuhn et al., 2021) as well as (class) identity dynamics (Sczepanski, 2023). Moreover, there is significant variation across countries. Institutions seem to matter here: research suggests that European identity tends to be more prominent among citizens of countries that have higher levels of institutional integration into the EU (Luhmann, 2017), and are among the countries using the Euro (Negri et al., 2021).

### The double-edged nature of European identity

Haas’ insistence on the integrating power of nationalism appears overly optimistic. Haas expected liberal nationalism to be the most promising basis for the international community (Haas, 1997, 19). However, he underestimated the polarizing potential of national identity in current European politics. Over the past decades, European integration and (de Wilde & Zürn, 2012; Hooghe & Marks, 2009; Hutter et al., 2016), and more generally, international authority (de Vries et al., 2021) has become highly politicized. Political entrepreneurs, most notably radical-right wing parties (Halikiopoulou et al., 2012), have been mobilizing exclusive national identities against European integration, despite overall increases in European identity. Hence, while Haas expected the public to enable further integration by pushing for supranational governance to maximize their material interests, the public today acts as a ‘constraining dissensus’ (Hooghe & Marks, 2009) that has the potential to slow down the speed and alter the direction of European integration.

This being said, when we take Haas’ argument about the two sides of nationalism on a more abstract level, it is insightful and serves as a powerful reminder that collective identity can be inclusive or exclusive. Whether it is inclusive or exclusive depends more on the content rather than the level (national vs supranational) of aggregation.

Without any doubt, the slow but steady growth of a feeling of European identity among the European public is good news. In Easton’s terms, European identity can serve as a reservoir of support also in crisis times when there is less motivation for specific, more policy-output oriented support (Easton, 1975). European identity has many positive behavioural and attitudinal correlates. European identifiers are more likely to show solidarity towards other Europeans (Bauhr & Charron, 2020; Nicoli et al., 2020; Verhaegen, 2018), openness towards immigrants (Curtis, 2014) and towards diverging political views (Stoeckel & Ceka, 2023).

However, while Europe’s violent past – the two world wars and the totalitarian regimes and political actors connected to them – was long seen as Europe’s Common Other, the perceptions are changing. The cordon sanitaire against political parties associated with extreme right and authoritarian positions is crumbling, as Ursula von der Leyen’s willingness to cooperate with Giorgia Meloni and her post-fascist party *Brothers of Italy* has shown (Jansen & Nguyen, 2024). Instead, new external and internal boundaries and Common Others are established, and far-right leaders such as Giorgia Meloni, Geert Wilders and Marine le Pen are trying to shape a new, rightwing narrative about Europe (Lorimer, 2020).

Risse (2010, p. 61) argued that next to the ‘modern’, cosmopolitan vision of Europe, a competing vision of Europe based on nationalist ideas exists. At the time that Risse wrote his book, the dividing lines between the modern and the nationalist vision seemed pretty clear, but they have become increasingly blurred and both visions are engaged by actors across the political spectrum. The nationalist vision of Europe is most obvious in illiberal narratives and ideologies that use Europe to bolster an illiberal nationalism writ large, such as the German PEGIDA^6^ or the motto ‘Make Europe Great Again’ (echoing Donald Trump’s ‘Make America Great Again’) of the Hungarian Presidency of the EU in 2024. Nationalist rhetoric and policy is also embedded in a pro-European, seemingly cosmopolitan narrative, as exemplified by the ‘Fortress Europe’.

These examples underline that European identity is not automatically an antidote to illiberal nationalism. When trying to overcome national egotisms and competition by creating a strong European identity, European policymakers might end up falling into the pitfalls of nationalism that they sought to overcome: fostering the idea that international politics is always a zero-sum game between ‘us’ vs ‘them’, establishing hard external borders and prompting feelings of superiority and hostility towards members of so-called Third Countries (Kuhn, 2023; Powers, 2022).

In this context, also collective identity building against Putin’s Russia as a Common Other bears its risks. In response to Russia’s invasion of Ukraine in 2013 and 2022, European citizens have ‘rallied round the flag’ and European identity and public support for the EU have increased (Gehring, 2022; Nicoli et al., 2024: Truchlewski et al., 2023). Hence, unwittingly, Putin might have contributed more to European unification than all efforts of EU leaders together because the invasion represents an existential security threat which is essential to state and nation-building (Kelemen & McNamara, 2022). However, Putin’s aggression has not only strengthened European identity, it also changed its content and the core values that are associated with it. In the wake of international conflict, many policymakers and citizens redefine what it means to be European, and change the narrative of a peaceful community built on European identity ‘lite’ to a more exclusive and belligerent one (Kuhn, 2023). While this is understandable and to a certain extent hard to avoid, it does bear the risk of creating an illiberal nationalism writ large.

In addition to hardening external borders, European identity-building can also lead to internal tensions and boundaries. Following Haas’ discussion on the struggles between competing national ideologies that lead to internal conflict (Haas, 1997, p. 43), the attempt to fuse different narratives about what the EU stands for into one unified narrative bears the risk of alienating and excluding those who do not fit into this narrative. As Powers (2022, p. 13) puts it, “The notion that supranationalism suppresses conflict ignores the fact that such broadly inclusive identities can paradoxically magnify intragroup animosity towards fellow regional residents who depart from the mold”.

In fact, regional and cultural hierarchies within the EU express themselves in a constant struggle over what makes a ‘good’ European. For instance, in the wake of the Euro crisis, a conflict emerged between Northern and Southern member states. Greece and other member states in severe economic difficulties needed economic rescue packages and in exchange implemented austerity policies (Biten et al., 2023). These predominantly Southern member states were portrayed as ‘lazy’ (Adler-Nissen, 2017) ‘sinners’ (Matthijs & McNamara, 2015) that needed to be ‘taught a lesson’ (Rathbun et al., 2019), while the EU and Northern member states such as Germany vilified as ‘Nazis’ colonizing the South (MacmiIlan, 2014).

## Conclusion

How insightful is Ernst B. Haas’ writing on nationalism for current research on European identity, and to what extent have his expectations about the relationship between national and supranational identity been supported by recent empirical studies? This paper set out to answer these questions by discussing the main arguments of Ernst B. Haas’ work on nationalism and contrasting it with the state of the art of European identity formation.

In his work on liberal nationalism, Haas broadened the focus from elites to citizens, and shifted from regional integration to liberal nationalism as a basis for the international community. He expected national and supranational identity to reflect a rational choice, and citizens to ultimately outgrow their national identities for utilitarian motivations. Haas also highlighted that not so much the level (national or supranational) of identity matters but rather its character – a liberal and inclusive nationalism can serve as basis for the international community.

Most importantly, there is a stark contrast between Haas’ conceptualization of collective identity as an inherently rational and deliberate choice on the one hand and more recent, empirically informed research that pictures collective identity as subliminal and emotional, resulting from a complex process. This contrast might help explain why Haas’s optimistic expectation of liberal nationalism as a groundswell of ‘international happiness’ did not pan out in reality. Rather than ‘outgrowing’ their nationalism and adopting a supranational outlook, many Europeans cling to their national allegiances and harbour hostile feelings towards non-nationals. Moreover, political entrepreneurs exploit these emotions and have successfully mobilized against European integration.

This being said, Haas’ insistence on the varieties of nationalism, and his preference for a liberal and inclusive nationalism is insightful for policymakers working on strengthening European identity. Haas reminds us that collective identity is double-edged: it can unify different groups but also divide and exclude. Hence, European policymakers should be careful not replace national identities and rather aim for a European identity ‘lite’. Internally, a European identity that replaces national identities weakens or even eliminates the positive aspects of national identity that Haas has been highlighting in his books (Haas, 1997, 2000). In Haas’ view, liberal nationalism provides an important social fabric and can unite diverging groups and their competing interests. Hence, European identity should be constructed as a supplement to national identity rather than in competition to it to safeguard the fabric that national communities are built on. Externally, it is important to steer clear from narratives of an exclusive European identity that competes with national identity as this bears the risk of replicating the fallacies of illiberal nationalism: Building a regional block with hard external borders, zero-sum reasoning and claims of superiority and hostility towards people and nations who are not perceived as European (Kuhn, 2023).

All in all, it seems that Haas was a rather stubborn optimist who did not want to give up his hope for ‘international happiness’. In his work on nationalism, Haas echoes his neofunctionalist approach by conceptualizing human actors as rational and deliberate, and he continues to be optimistic about the potential for human and societal progress. It seems what has changed most throughout Haas’ career is the emphasis on the political level of aggregation. While in his neofunctionalist work on regional integration, Haas put all his bets on supranational institutions, in his later work he focuses on the national community as the main agent of change. Moreover, while Haas found it safe to ignore citizen identities and preferences in his early work on regional integration, mass politics plays a more central role in his work on liberal nationalism.

With benefit of hindsight, Haas seems to have been overly optimistic, and his expectations about rationality as a source for identity appear somewhat simplistic. Collective identities are not switched off and on, nationalists cannot ‘easily be paid off', as he asserted, and political entrepreneurs continue to mobilize collective identities against international cooperation. However, where would Europe be today, without its visionaries? Haas’ optimism, even if it was far-fetched, might have been able to bring us closer to his vision of ‘international happiness’ in Europe by inspiring and encouraging policymakers to strive for regional cooperation.

## Notes

1 Ironically, a few years after he did so, the Single European Act was passed and European integration was relaunched in something that looked very neofunctionalist.

2 This changed as the European Parliament increasingly became akin to a “lower chamber”, the EP elections have some influence on the makeup of the European commission, and through the increasing use of referendums.

3 This this the title of one of the chapters in *Nationalism, Liberalism and Progress*, referring to Haas’ conception of nationalism (Haas 1997: 22).

4 To be sure, there might be also processes of deliberate identity construction that are not based on rationalist utility calculations. Abiding by certain norms and adopting a certain identity can under certain circumnstances also be the result of a deliberate choice to belong to a certain community, following a logic of appropriateness rather than a logic of consequences.

5 The question has been asked since 1992. For consistency, only data from 1995 onwards are shown as not all EU-15 were surveyed before 1995. The wording of the question has been subject to change, but the most common wording is as follows: ‘In the near future, do you see yourself as only (nationality), (nationality) and European, European and (nationality), European only’. In 2019, the following question was asked: ‘You feel you are a citizen of the EU’ (answer categories definitely, to some extent, not really, definitely not), dichotomized into definitely/some extent vs the rest. As only small minorities see themselves as European first, or European only, observations were dichotomized into exclusive nationalists and respondents (also) identifying as European.

6 Patriotic Europeans against the Islamization of the Occident.

## References

[CIT0001] Abdelal, R., Herrera, Y. M., Johnston, A. I., & McDermott, R. (2006). Identity as a variable. *Perspectives on Politics*, *4*(4), 695–711. 10.1017/S1537592706060440

[CIT0002] Adler-Nissen, R. (2017). Are we ‘lazy Greeks’ or ‘Nazi Germans’? *Hierarchies in World Politics*, *144*, 198–218. 10.1017/9781108241588.011

[CIT0003] Anderson, B. (1983). *Imagined communities: Reflections on the origin and spread of nationalism*. Verso.

[CIT0004] Bauhr, M., & Charron, N. (2020). In God we trust? Identity, institutions and international solidarity in Europe. *JCMS: Journal of Common Market Studies*, *58*(5), 1124–1143. 10.1111/jcms.13020

[CIT0005] Bergbauer, S. (2018). *Explaining European identity formation*. Springer.

[CIT0006] Biten, M., Kuhn, T., & van der Brug, W. (2023). How does fiscal austerity affect trust in the European Union? Analyzing the role of responsibility attribution. *Journal of European Public Policy*, *30*(6), 1033–1050. 10.1080/13501763.2022.2060282

[CIT0007] Börzel, T. A. (2024). Regionalism and liberal nationalism in the European Union. A case Sui Generis? *Journal of European Public Policy*, 1–22. 10.1080/13501763.2024.2321987

[CIT0008] Brooks, E., de Ruijter, A., Greer, S. L., & Rozenblum, S. (2023). EU health policy in the aftermath of COVID-19: Neofunctionalism and crisis-driven integration. *Journal of European Public Policy*, *30*(4), 721–739. 10.1080/13501763.2022.2141301

[CIT0009] Brubaker, R., & Laitin, D. D. (1998). Ethnic and nationalist violence. *Annual Review of Sociology*, *24*(1), 423–452. 10.1146/annurev.soc.24.1.423

[CIT0010] Bruter, M. (2003). Winning hearts and minds for Europe. *Comparative Political Studies*, *36*(10), 1148–1179. 10.1177/0010414003257609

[CIT0011] Caramani, D. (2024). Community and governance beyond the nation-state in the 21st century: Introduction to the special issue on the legacy of Ernst B. Haas. *Journal of European Public Policy*, 1–7. 10.1080/13501763.2024.2314243

[CIT0012] Checkel, J. T. (2005). International institutions and socialization in Europe: Introduction and framework. *International Organization*, *59*(4), 801–826. 10.1017/S0020818305050289

[CIT0013] Checkel, J. T., & Katzenstein, P. J. 2009). *European identity*. Cambridge University Press.

[CIT0014] Cram, L. (2012). Does the EU need a navel? Implicit and explicit identification with the European Union*. *Jcms: Journal of Common Market Studies*, *50*(1), 71–86. 10.1111/j.1468-5965.2011.02207.x

[CIT0015] Cram, L., & Patrikios, S. (2015). Visual primes and European Union identity: Designing experimental research. In K. Lynggaard, I. Manners, & K. Löfgren (Eds.), *Research methods in European studies* (pp. 184–205). Palgrave Macmillan.

[CIT0016] Curtis, K. A. (2014). Inclusive versus exclusive: A cross-national comparison of the effects of subnational, national, and supranational identity. *European Union Politics*, *15*(4), 521–546. 10.1177/1465116514528058

[CIT0017] Dennison, J., Davidov, E., & Seddig, D. (2020). Explaining voting in the UK's 2016 EU referendum: Values, attitudes to immigration, European identity and political trust. *Social Science Research*, *92*, 102476. 10.1016/j.ssresearch.2020.102476 33172565

[CIT0018] Deutsch, K. W. (1954 [1970]). *Political community at the international level. Problems of definition and measurement*. Doubleday & Company, Inc.

[CIT0019] Deutsch, K. W., Burrel, S. A., Kann, R. A., Lee, M. Jr., Lichterman, M., Lindgren, R. E., Loewenheim, F. L., & Van Wagenen, R. W. (1957). *Political Community and the North Atlantic Area*. Greenwood Press, Publishers.

[CIT0020] Deutschmann, E., Delhey, J., Verbalyte, M., & Aplowski, A. (2018). The power of contact: Europe as a network of transnational attachment. *European Journal of Political Research*, *57*(4), 963–988. 10.1111/1475-6765.12261

[CIT0021] De Vries, C. E. (2018). *Euroscepticism and the future of European integration*. Oxford University Press.

[CIT0022] De Vries, C. E., Hobolt, S. B., & Walter, S. (2021). Politicizing international cooperation: The mass public, political entrepreneurs, and political opportunity structures. *International Organization*, *75*(2), 306–332. 10.1017/S0020818320000491

[CIT0023] De Wilde, P., & Zürn, M. (2012). Can the politicization of European integration be reversed?*. *JCMS: Journal of Common Market Studies*, *50*(s1), 137–153. 10.1111/j.1468-5965.2011.02232.x

[CIT0024] Easton, D. (1975). A re-assessment of the concept of political support. *British Journal of Political Science*, *5*(4), 435–457. 10.1017/S0007123400008309

[CIT0025] Fernández, J. J., & Eigmüller, M. (2018). Societal education and the education divide in European identity, 1992–2015. *European Sociological Review*, *34*(6), 612–628. 10.1093/esr/jcy031

[CIT0026] Fernández, J. J., Teney, C., & Díez Medrano, J. (2023). Mechanisms of the effect of individual education on pro-European dispositions. *Journal of Common Market Studies*, 1–22.

[CIT0027] Fligstein, N. (2008). *Euroclash: The EU, European identity, and the future of Europe*. Oxford University Press.

[CIT0028] Gehring, K. (2022). Can external threats foster a European Union identity? Evidence from Russia’s invasion of Ukraine. *The Economic Journal*, *132*(644), 1489–1516. 10.1093/ej/ueab088

[CIT0029] Haas, E. B. (1958). *The uniting of Europe: Political, social, and economic forces, 1950–1957*. University of Notre Dame Press.

[CIT0030] Haas, E. B. (1964). *Beyond the nation-state : Functionalism and international organization*. Stanford University Press.

[CIT0031] Haas, E. B. (1970). The study of regional integration: Reflections on the joy and anguish of pretheorizing. *International Organization*, *24*(4), 606–646. 10.1017/S0020818300017495

[CIT0032] Haas, E. B. (1976). Turbulent fields and the theory of regional integration. *International Organization*, *30*(2), 173–212. 10.1017/S0020818300018245

[CIT0033] Haas, E. B. (1986). What is nationalism and why should we study it? *International Organization*, *40*(3), 707–744. 10.1017/S0020818300027326

[CIT0034] Haas, E. B. (1997). *Nationalism, liberalism, and progress: The rise and decline of nationalism* (Vol. 1). Cornell University Press.

[CIT0035] Haas, E. B. (2000). *Nationalism, liberalism, and progress: The dismal fate of new nations* (Vol. 2). Cornell University Press.

[CIT0036] Hadler, M., Chin, L., & Tsutsui, K. (2021). Conflicting and reinforcing identities in expanding Europe from 1995 to 2019. Findings revisited in an even larger Europe. *Innovation: The European Journal of Social Science Research*, *34*(1), 3–13. 10.1080/13511610.2020.1745060

[CIT0037] Halikiopoulou, D., Nanou, K., & Vasilopoulou, S. (2012). The paradox of nationalism: The common denominator of radical right and radical left euroscepticism. *European Journal Of Political Research*, *51*(4), 504–539. 10.1111/j.1475-6765.2011.02050.x

[CIT0038] Hechter, M. (2000). *Containing nationalism*. Oxford University Press.

[CIT0039] Hobolt, S. B. (2016). The Brexit vote: A divided nation. *Journal of European Public Policy*, *23*, 1259–1277.

[CIT0040] Hoffmann, S. (1966). Obstinate or obsolete? The fate of the nation-state and the case of Western Europe. *Daedalus*, *95*(3), 862–915.

[CIT0041] Hooghe, L. (2005). Several roads lead to international norms, but few via international socialization: A case study of the European commission. *International Organization*, *59*(4), 861–898.

[CIT0042] Hooghe, L., & Marks, G. (2009). A postfunctionalist theory of European integration: From permissiveconsensus to constraining dissensus. *British Journal of Political Science*, *39*(1), 1–23. 10.1017/S0007123408000409

[CIT0043] Hooghe, L., & Marks, G. (2018). Cleavage theory meets Europe’s crises: Lipset, Rokkan, and the transnational cleavage. *Journal of European Public Policy*, *25*(1), 109–135. 10.1080/13501763.2017.1310279

[CIT0044] Hooghe, L., & Marks, G. (2019). Grand theories of European integration in the twenty-first century. *Journal of European Public Policy*, *26*(8), 1113–1133. 10.1080/13501763.2019.1569711

[CIT0045] Huddy, L., & Del Ponte, A. (2019). National identity, pride, and chauvinism-their origins and consequences for globalization attitudes. *Liberal Nationalism and Its Critics*, *12*(2), 38–56.

[CIT0046] Hutter, S., Grande, E., & Kriesi, H. (2016). *Politicising Europe*. Cambridge University Press.

[CIT0047] Jansen, J., & Nguyen, T. (2024). Between continuity and a perforated ‘cordon sanitaire’ On the 2024 European elections. Policy brief, Jacques Delors centre. https://www.delorscentre.eu/fileadmin/2_Research/1_About_our_research/2_Research_centres/6_Jacques_Delors_Centre/Publications/20240613_On_the_2024_European_Elections_Nguyen_Jansen.pdf.

[CIT0048] Jung, J. K. (2008). Growing supranational identities in a globalising world? A multilevel analysis of the world values surveys. *European Journal of Political Research*, *47*(5), 578–609. 10.1111/j.1475-6765.2008.00779.x

[CIT0049] Kaina, V., & Karolewski, I. P. (2013). EU governance and European identity. *Living Reviews in European Governance*, *8*(13), 1–59.

[CIT0050] Kaufmann, E. (2019). Can narratives of white identity reduce opposition to immigration and support for hard brexit? A survey experiment. *Political Studies*, *67*(1), 31–46. 10.1177/0032321717740489

[CIT0051] Kelemen, R. D., & McNamara, K. R. (2022). State-building and the European Union: Markets, War, and Europe’s uneven political development. *Comparative Political Studies*, *55*(6), 963–991.

[CIT0052] Kohli, M. (2000). The battlegrounds of European identity. *European Societies*, *2*(2), 113–137. 10.1080/146166900412037

[CIT0053] Kohn, H. (1944). *The idea of nationalism: A study in its origins and background*. Macmillan.

[CIT0054] König, P. (2023). Forms of national and European identity: A research note reviewing literature of cross-national studies. *Nationalities Papers*, 1–28. 10.1017/nps.2023.66

[CIT0055] Kriesi, H., Grande, E., Lachat, R., Dolezal, M., Bornschier, S., & Frey, T. (2008). *West European politics in the age of globalization*. Cambridge University Press.

[CIT0056] Kuhn, T. (2015). *Experiencing European integration: Transnational lives and European identity*. OUP.

[CIT0057] Kuhn, T. (2019). Grand theories of European integration revisited: Does identity politics shape the course of European integration? *Journal of European Public Policy*, *26*(8), 1213–1230. 10.1080/13501763.2019.1622588

[CIT0058] Kuhn, T. (2023). Common institutions, diverging identities? Supranational institution building and collective identity formation in the European Union. *Inaugural lecture*, University of Amsterdam. https://dare.uva.nl/search?identifier=f4940a94-41c3-4770-8901-e6cb20114648

[CIT0059] Kuhn, T., Lancee, B., & Sarrasin, O. (2021). Growing up as a European? Parental socialization and the educational divide in Euroskepticism. *Political Psychology*, *42*(6), 957–975. 10.1111/pops.12728

[CIT0060] Lorimer, M. (2020). Europe as ideological resource: The case of the Rassemblement National. *Journal of European Public Policy*, *27*(9), 1388–1405. 10.1080/13501763.2020.1754885

[CIT0061] Luhmann, S. (2017). A multi-level approach to European identity: Does integration foster identity? *JCMS: Journal of Common Market Studies*, *55*(6), 1360–1379. 10.1111/jcms.12554

[CIT0062] Lutz, W., Kritzinger, S., & Skirbekk, V. (2006). The demography of growing European identity. *Science*, *314*(5798), 425–425. 10.1126/science.1128313 17053133

[CIT0063] Macmillan, C. (2014). The return of the Reich? A Gothic tale of Germany and the Eurozone crisis. *Journal of Contemporary European Studies*, *22*(1), 24–38. 10.1080/14782804.2014.887891

[CIT0064] Matthijs, M., & McNamara, K. (2015). The euro crisis’ theory effect: Northern saints, southern sinners, and the demise of the Eurobond. *Journal of European Integration*, *37*(2), 229–245. 10.1080/07036337.2014.990137

[CIT0065] McNamara, K. R. (2015). *The politics of everyday Europe: Constructing authority in the European Union*. Oxford University Press.

[CIT0066] Milner, H. V. (1998). Rationalizing politics: The emerging synthesis of international, American, and comparative politics. *International Organization*, *52*(4), 759–786. 10.1162/002081898550743

[CIT0067] Negri, F., Nicoli, F., & Kuhn, T. (2021). Common currency, common identity? The impact of the Euro introduction on European identity. *European Union Politics*, *22*(1), 114–132. 10.1177/1465116520970286

[CIT0068] Nicoli, F. (2020). Neofunctionalism revisited: Integration theory and varieties of outcomes in the Eurocrisis. *Journal of European Integration*, *42*(7), 897–916. 10.1080/07036337.2019.1670658

[CIT0069] Nicoli, F., Kuhn, T., & Burgoon, B. (2020). Collective identities, European solidarity: Identification patterns and preferences for European social insurance. *JCMS: Journal of Common Market Studies*, *58*(1), 76–95. 10.1111/jcms.12977

[CIT0070] Nicoli, F., van der Duin, D., Beetsma, R., Bremer, B., Burgoon, B., Kuhn, T., Meijers, M., & de Ruiter, A. (2024). Closer during crises? European identity during the COVID-19 pandemic and the Russian invasion of Ukraine. *Journal of European Public Policy*, 1–27. 10.1080/13501763.2024.2366395

[CIT0071] Norris, P. (2000). Global governance and cosmopolitan citizens. In J. S. Nye & J. D. Donahue (Eds.), *Governance in a globalizing world* (pp. 155–175). Brookings Institution Press.

[CIT0072] Powers, K. E. (2022). *Nationalisms in international politics*. Princeton University Press.

[CIT0073] Rathbun, B. C., Powers, K. E., & Anders, T. (2019). Moral Hazard: German public opinion on the Greek debt crisis. *Political Psychology*, *40*(3), 523–541. 10.1111/pops.12522

[CIT0074] Reinl, A. K., Nicoli, F., & Kuhn, T. (2023). Regional inequalities and transnational solidarity in the European Union. *Political Geography*, *104*, 102903. 10.1016/j.polgeo.2023.102903

[CIT0075] Rekker, R. (2018). Growing up in a globalized society: Why younger generations are more positive about the European Union. *Young*, *26*(4_suppl), 56S–77S. 10.1177/1103308817748433 30595644 PMC6294187

[CIT0076] Risse, T. (1998). In defense of liberal nationalism. *Mershon International Studies Review*, *42*(1), 177–179. 10.2307/254462

[CIT0077] Risse, T. (2010). *A European community? Transnational identities and public spheres*. Cornell University Press.

[CIT0078] Rosamond, B. (2005). The uniting of Europe and the foundation of EU studies: Revisiting the neofunctionalism of Ernst B. Haas. *Journal of European Public Policy*, *12*(2), 237–254. 10.1080/13501760500043928

[CIT0079] Ruggie, G. J., Katzenstein, P. J., Keohane, R. O., & Schmitter, P. C. (2005). Transformations in world politics: The intellectual contributions of Ernst B. *Annual Review of Political Science*, *8*(1), 271–296. 10.1146/annurev.polisci.8.082103.104843

[CIT0080] Saurugger, S., & Thatcher, M. (2019). Constructing the EU’s political identity in policy making. *Comparative European Politics*, *17*(4), 461–476. 10.1057/s41295-019-00169-2

[CIT0081] Schimmelfennig, F. (2016). A differentiated leap forward: Spillover, path-dependency, and graded membership in European banking regulation. *West European Politics*, *39*(3), 483–502. 10.1080/01402382.2016.1143244

[CIT0082] Schlenker, A. (2013). Cosmopolitan Europeans or partisans of fortress Europe? Supranational identity patterns in the EU. *Global Society*, *27*(1), 25–51. 10.1080/13600826.2012.734281

[CIT0083] Schröder, M., Braun, D., Grimm, K. L., Ulrich, M., & Wenzelburger, G. (2024). No European generations, but EU-attachment increases over time. An age period cohort analysis of emotional attachment to the EU. *Journal of European Public Policy*, 1–29.

[CIT0084] Sczepanski, R. (2023). Who are the cosmopolitans? How perceived social sorting and social identities relate to European and national identities. *Comparative Political Studies*, 57(7), 1210–1239.

[CIT0085] Shorrocks, R., & De Geus, R. (2019). Citizen support for European Union membership: The role of socialisation experiences. *West European Politics*, *42*(4), 873–894. 10.1080/01402382.2018.1560199

[CIT0086] Stoeckel, F., & Ceka, B. (2023). Political tolerance in Europe: The role of conspiratorial thinking and cosmopolitanism. *European Journal of Political Research*, *62*(3), 699–722. 10.1111/1475-6765.12527

[CIT0087] Tajfel, H. (1982). Social psychology of intergroup relations. *Annual Review of Psychology*, *33*(1), 1–39. 10.1146/annurev.ps.33.020182.000245

[CIT0088] Truchlewski, Z., Oana, I. E., & Moise, A. D. (2023). A missing link? Maintaining support for the European polity after the Russian invasion of Ukraine. *Journal of European Public Policy*, *30*(8), 1662–1678. 10.1080/13501763.2023.2218419

[CIT0089] Verhaegen, S. (2018). What to expect from European identity? Explaining support for solidarity in times of crisis. *Comparative European Politics*, *16*(5), 871–904. 10.1057/s41295-017-0106-x

[CIT0090] Zeitlin, J., Nicoli, F., & Laffan, B. (2019). Introduction: The European Union beyond the polycrisis? Integration and politicization in an age of shifting cleavages. *Journal of European Public Policy*, *26*(7), 963–976. 10.1080/13501763.2019.1619803

